# Estimating probability of germline mismatch repair mutations in colorectal cancer patients with microsatellite stable tumors

**DOI:** 10.1186/1897-4287-9-S1-P12

**Published:** 2011-03-10

**Authors:** Jessica N Everett, Victoria M Raymond, Michele Gornick, Rajesh S Mangrulkar, Ignacio Blanco, Stephen B Gruber

**Affiliations:** 1 Department of Internal Medicine, University of Michigan, Ann Arbor, Michigan, 48109, USA; 2 Department of Human Genetics, University of Michigan, Ann Arbor, Michigan, 48109, USA; 3 Cancer Genetic Counseling Program, IDIBELL-Institut Català d’Oncologia, Barcelona, 08907, Spain; 4 Department of Internal Medicine, Epidemiology, and Human Genetics, University of Michigan, Ann Arbor, Michigan, 48109, USA

## Background

Microsatellite instability (MSI) is a hallmark of DNA mismatch repair (MMR) deficiency and is an established screening tool for identifying Lynch syndrome in colorectal cancer populations [1]. However, MSI testing is neither perfectly sensitive nor specific to detect Lynch Syndrome, and germline MMR mutations have been reported in patients with microsatellite stable (MSS) tumors [2]. The value of germline MMR testing in patients with MSS tumors may vary based on family history, and data is needed to guide choices about when to offer testing in high risk clinic settings.

## Materials and methods

From 2002-2009, high risk patients presenting to the Cancer Genetics Clinics at the University of Michigan and the Catalan Institute of Oncology in Barcelona, Spain were evaluated for Lynch syndrome and included in the present study. Patients with MSS colorectal tumors who also had germline MMR testing were eligible for analysis. We calculated risk for MMR mutation using MMRPro v.5.1[3] at baseline, after MSI testing, and after germline MMR testing. Modified likelihood ratios were estimated to evaluate the utility of germline testing in patients with MSS tumors based on the strength of the family history [4].

## Results

Germline MMR mutations were identified in 5/44 (11.4%) patients with MSS tumors. Two of the mutations were identified in patients from families that met the Amsterdam Criteria (AC I/II), whereas 3 mutations were found in patients from families that were AC I/II negative. The modified likelihood for a germline mutation (+LR_mod_) in an AC I/II patient was 1.56 (95% CI: 0.47 - 5.18) and the modified negative likelihood ratio (-LR_mod_) for a patient not meeting the AC I/II criteria was 0.81 (95% CI: 0.39 - 1.69). We quantified the meaning of the AC I/II criteria to guide clinical choices about genetic testing in MSS tumors by multiplying the pretest odds by the +LR_mod_ and the -LR_mod_. The probability of a mutation was modified from a baseline of 11.4% to 16.7% in AC I/II families, compared to 9.4% in AC I/II-negative families.

## Conclusions

Germline MMR testing in high risk patients with MSS colorectal tumors identifies mutations in a small, but meaningful proportion of patients. The diagnostic yield is dependent on the strength of the family history. Modified likelihood ratios can be helpful to quantify the probability of a positive gene test for Lynch Syndrome, and can be applied to pre-test probabilities derived from clinical models.
